# Effect of AI-based chatbots in promoting oral health awareness among rural populations

**DOI:** 10.6026/973206300210827

**Published:** 2025-04-30

**Authors:** Rohit Agrawal, Asim Mustafa Khan, Muhamood Moothedath, Reshma V.J, Muhaseena Muhamood, Shivangee T

**Affiliations:** 1 Department of Public Health Dentistry, Government Dental College and Hospital, Jamnagar, Gujarat, India; 2 Department of Biomedical dental sciences, College of dentistry, Imam Abdulrahman Bin Faisal University, Dammam, Saudi Arabia; 3 Department of Public Health, College of Applied Medical Sciences, Qassim University, Saudi Arabia; 4 Department of Dental Surgery, MBA in Healthcare Administration at City University of Seattle, Seattle, Washington, USA

**Keywords:** AI-based chatbot, oral health awareness, rural population, health education, artificial intelligence, digital health intervention

## Abstract

AI-based chatbots significantly improved oral health awareness among rural populations compared to traditional printed materials. The intervention group's knowledge scores increased from 8.5 to 16.7 (p < 0.001), while the control group showed a modest rise from 8.3 to 10.5. Inter-group analysis confirmed a significant knowledge gain (p < 0.001). Participants reported high satisfaction with the chatbot's personalized and user-friendly interface. Thus, AI chatbots offer a promising solution for scalable, accessible oral health education in underserved communities.

## Background:

Oral health is a fundamental aspect of overall well-being and quality of life, yet it remains a neglected area of public health, especially in rural and underserved populations. Poor oral health is associated with systemic conditions such as cardiovascular disease, diabetes and adverse pregnancy outcomes, emphasizing the need for effective preventive strategies [1, 2]. Despite this, barriers such as limited access to dental care, low literacy levels and lack of awareness contribute to a higher prevalence of oral diseases in rural areas [3]. In recent years, digital health technologies have gained prominence as cost-effective and scalable solutions for addressing health disparities. Among these, Artificial Intelligence (AI)-based chatbots have emerged as innovative tools for delivering personalized health information and promoting behavioral change [4, 5]. Chatbots are capable of engaging users in interactive, conversational exchanges, making them particularly effective in regions with limited access to traditional healthcare services [6]. Application of AI chatbots is an emerging field of research in lifestyle modification programs and is expected to grow exponentially [7, 8]. However, there is limited research on the application of AI-based chatbots in oral health promotion, particularly in rural settings where they could address key challenges such as resource constraints and geographic isolation. This study aims to evaluate the effectiveness of an AI-based chatbot in enhancing oral health awareness among rural populations. By comparing its impact to traditional methods, this research seeks to provide evidence for integrating AI technologies into oral health education programs in underserved areas. In rural and underserved communities, oral health often takes a back seat due to more pressing health concerns and resource constraints. This neglect is exacerbated by a lack of awareness about the importance of oral hygiene and its impact on systemic health. For instance, untreated periodontal diseases can lead to inflammation that contributes to conditions such as cardiovascular disease and diabetes [9].

AI chatbots have demonstrated the efficacy of health behavior change interventions among large and diverse populations [10]. These findings underscore the importance of proactive oral health education and preventive strategies, particularly in areas where access to professional dental care is limited. AI-based chatbots provide a promising avenue for overcoming the traditional barriers to oral health education. Unlike conventional methods, chatbots are interactive, customizable and available 24/7, making them highly accessible even in remote areas. These tools leverage natural language processing and machine learning to deliver tailored health information, which can address the specific needs of diverse populations [11]. For example, a chatbot designed for a rural population could provide guidance in the local language and focus on common oral health issues in that demographic. Furthermore, the use of AI chatbots can reduce the dependency on physical infrastructure and human resources, making them a cost-effective solution for large-scale health education programs [12]. Despite these advantages, the integration of AI technologies into oral health promotion faces several challenges. A key concern is digital literacy, which remains low in many rural populations. Although smartphone penetration is increasing, a significant proportion of individuals may not feel comfortable navigating apps or understanding the chatbot's functionality [13]. Additionally, ensuring the cultural relevance of the chatbot's content is crucial for its acceptance and effectiveness. For instance, dietary habits and traditional oral health practices vary widely across regions and must be considered when designing chatbot interventions [14]. Addressing these challenges requires collaboration between technology developers, healthcare providers and community leaders to create solutions that are user-friendly, culturally appropriate and impactful. Therefore, it is of interest to assess the effect of AI-based chatbots in promoting oral health awareness among rural populations.

## Materials and Methods:

## Study design:

This was a cross-sectional study conducted over three months to evaluate the effectiveness of an AI-based chatbot in improving oral health awareness among rural populations. Ethical approval for the study was obtained from the institutional ethics committee and informed consent was secured from all participants.

## Study population:

A total of 500 participants aged 18-60 years were recruited from rural areas using convenience sampling. Inclusion criteria included individuals who could read and understand the chatbot interface language and had access to a smartphone. Exclusion criteria included those with prior exposure to similar digital health tools or professional oral health education within the past six months.

## Intervention and control groups:

Participants were divided into two groups of 250 each. The intervention group interacted with an AI-based chatbot designed to provide oral health education, including information on dental hygiene practices, common oral diseases and preventive measures. The chatbot also answered users' questions and provided personalized recommendations based on user input. The control group received traditional oral health education via printed materials, such as brochures and posters, detailing similar topics.

## Study tool:

A validated 20-item multiple-choice questionnaire was used to assess participants' oral health knowledge before and after the intervention. The questionnaire covered topics such as dental hygiene, the effects of diet on oral health and the importance of regular dental check-ups. The content validity of the questionnaire was reviewed by a panel of dental experts and a pilot test ensured its reliability.

## Procedure:

Baseline oral health knowledge was assessed for both groups using the questionnaire. The intervention group interacted with the chatbot for four weeks, with participants encouraged to use the chatbot at least three times per week. The control group received the printed materials at the beginning of the study. After four weeks, post-intervention knowledge was assessed using the same questionnaire.

## Data analysis:

Data were analysed using SPSS version 25.0. Paired t-tests were performed to compare pre- and post-intervention knowledge scores within each group, while independent t-tests were used to compare the mean score changes between groups. A p-value of <0.05 was considered statistically significant.

## Outcome measures:

The primary outcome was the change in oral health knowledge scores from baseline to post-intervention. Secondary outcomes included participant satisfaction with the chatbot and their willingness to recommend it to others.

## Results:

A total of 500 participants completed the study, with an equal distribution in the intervention group (n=250) and the control group (n=250). The demographic characteristics of participants, such as age, gender and education level, were comparable between the groups (Table 1). The intervention group showed a significant improvement in oral health knowledge scores, with the mean pre-intervention score increasing from 8.5 ± 2.1 to 16.7 ± 2.5 post-intervention (p < 0.001). The control group also demonstrated an increase in scores, but it was less pronounced, rising from 8.3 ± 2.0 to 10.5 ± 2.3 (p < 0.05). The mean difference in score improvement between the two groups was statistically significant (p < 0.001) (Table 2). Among the intervention group, 92% of participants reported satisfaction with the chatbot's functionality, while 85% expressed willingness to recommend the chatbot to others. Additionally, 78% of participants in the intervention group believed the chatbot was more engaging than traditional educational materials. The data in Table 1 confirms the comparability of baseline demographic characteristics between groups, ensuring minimal bias. The knowledge score improvements detailed in Table 2, Figure 1 (see PDF) further demonstrate the superior effectiveness of the AI-based chatbot in promoting oral health awareness compared to traditional educational materials.

## Discussion:

This study evaluated the effectiveness of an AI-based chatbot in improving oral health awareness among rural populations. The findings revealed that the intervention group showed a significant increase in oral health knowledge scores compared to the control group, indicating the potential of AI-driven tools to address health education gaps in underserved areas. The use of chatbots in health education aligns with the growing adoption of digital technologies in healthcare. AI-based chatbots can provide interactive, engaging and personalized learning experiences, which may explain their superior performance compared to static printed materials [1, 2]. Previous research has demonstrated that chatbots are effective in enhancing knowledge and behavior across various domains, including mental health, chronic disease management and smoking cessation [3, 4]. However, their application in oral health promotion remains underexplored. The significant improvement in knowledge scores among the intervention group suggests that chatbots can effectively communicate complex oral health concepts in an accessible manner. This is particularly important in rural areas, where low literacy levels and limited access to dental care are common barriers [5, 6]. Potential of AI chatbots in areas such as healthy lifestyle promotion, smoking cessation, medication adherence and reduction in substance misuse is booming day by day. Various AI applications, including personalized educational materials, virtual consultations, language translation tools and virtual reality simulations, which can enhance patient understanding and experience [7, 8]. Participant feedback also underscores the value of user-friendly interfaces and personalized recommendations. High satisfaction rates and willingness to recommend the chatbot indicate its acceptability in rural settings. Incorporating gamification and behavior change strategies, the chatbot aims to enhance children's engagement in oral hygiene practices [9, 10]. Additionally, the interactivity of chat bots may encourage behavior change, a critical component of preventive oral health strategies [11]. Despite the positive results, certain limitations should be considered. The study relied on self-reported data, which may be subject to social desirability bias. Furthermore, the short duration of the intervention limits the ability to assess long-term behavioral changes. Future studies should explore the sustained impact of chatbot-based education and its integration with existing healthcare systems [12, 13]. This study also raises important implications for public health policymakers. AI-based chatbots offer a cost-effective, scalable solution for delivering oral health education, particularly in resource-limited settings. Their integration into broader health promotion programs could bridge the gap between underserved populations and essential health information [14]. The findings of this study highlight the potential for AI-based chatbots to not only improve oral health knowledge but also to reduce disparities in access to health education. Rural and underserved populations often face systemic challenges, including geographic isolation and limited healthcare infrastructure. AI-based chatbots show promise in enhancing user engagement and facilitating personalized weight loss interventions by integrating behaviour change techniques, emotional cues and multi-platform interactivity [15]. Future research should also explore the integration of AI chatbots with telemedicine platforms to enhance their functionality and reach. For example, combining chatbot education with virtual dental consultations could provide a more holistic approach to oral health promotion, addressing both knowledge gaps and the need for professional guidance. Additionally, evaluating the cost-effectiveness of chatbot interventions compared to other educational strategies will be crucial in advocating for their widespread adoption. Addressing technical challenges, such as improving chatbot algorithms for better natural language understanding and ensuring data security, will further enhance their reliability and scalability in diverse settings.

## Conclusion:

AI-based chatbots are a promising tool for promoting oral health awareness in rural populations. They demonstrate significant advantages over traditional methods, including enhanced knowledge, user satisfaction and engagement. However, it is important to evaluate their long-term impact on oral health behaviors and outcomes.

## Figures and Tables

**Table 1:** Demographic characteristics of participants

**Characteristic**	**Intervention Group (n=250)**	**Control Group (n=250)**	**p-value**
Mean age (years)	35.2 ± 10.5	34.8 ± 9.8	0.65
Gender (M/F)	120/130	125/125	0.78
**Education Level (%)**			
- Primary school	45 (18%)	50 (20%)	0.72
- Secondary school	130 (52%)	125 (50%)	0.85
- Higher education	75 (30%)	75 (30%)	0.9

**Table 2:** Comparison of knowledge scores

**Group**	**Pre-Intervention Mean ± SD**	**Post-Intervention Mean ± SD**	**Mean Difference**	**p-value**
Intervention Group	8.5 ± 2.1	16.7 ± 2.5	8.2	<0.001
Control Group	8.3 ± 2.0	10.5 ± 2.3	2.2	<0.05
